# Tuning Properties of Partially Reduced Graphene Oxide Fibers upon Calcium Doping

**DOI:** 10.3390/nano10050957

**Published:** 2020-05-18

**Authors:** Krzysztof Tadyszak, Jacek K. Wychowaniec, Karol Załęski, Emerson Coy, Łukasz Majchrzycki, Raanan Carmieli

**Affiliations:** 1 Institute of Molecular Physics, Polish Academy of Sciences, ul. Smoluchowskiego 17, 60-179 Poznań, Poland; 2 School of Chemistry, University College Dublin, Belfield, Dublin 4, Ireland; jacek.wychowaniec@ucd.ie; 3 NanoBioMedical Centre, Adam Mickiewicz University, Wszechnicy Piastowskiej 3, 61-614 Poznań, Poland; zaleski@amu.edu.pl (K.Z.); coyeme@amu.edu.pl (E.C.); 4 Center of Advanced Technology, Adam Mickiewicz University, ul. Uniwersytetu Poznańskiego 10, 61-614 Poznań, Poland; lukmaj@amu.edu.pl; 5 Department of Chemical Research Support, Faculty of Chemistry, Weizmann Institute of Science, Rehovot 76100, Israel; raanan.carmieli@weizmann.ac.il

**Keywords:** graphene oxide fibers, hydrothermal synthesis, reduced graphene oxide, electron paramagnetic resonance, electron spin relaxation

## Abstract

The arrangement of two-dimensional graphene oxide sheets has been shown to influence physico-chemical properties of the final bulk structures. In particular, various graphene oxide microfibers remain of high interest in electronic applications due to their wire-like thin shapes and the ease of hydrothermal fabrication. In this research, we induced the internal ordering of graphene oxide flakes during typical hydrothermal fabrication via doping with Calcium ions (~6 wt.%) from the capillaries. The Ca^2+^ ions allowed for better graphene oxide flake connections formation during the hydrogelation and further modified the magnetic and electric properties of structures compared to previously studied aerogels. Moreover, we observed the unique pseudo-porous fiber structure and flakes connections perpendicular to the long fiber axis. Pulsed electron paramagnetic resonance (EPR) and conductivity measurements confirmed the denser flake ordering compared to previously studied aerogels. These studies ultimately suggest that doping graphene oxide with Ca^2+^ (or other) ions during hydrothermal methods could be used to better control the internal architecture and thus tune the properties of the formed structures.

## 1. Introduction

Graphene oxide (GO) is a form of graphene having a unique collection of oxygen rich groups on its surface (-COOH, -OH, =O, -O-) [1,2]. Because of oxygen rich moieties it is a promising material for sensing in biology and medicine: high-contrast bio-imaging and bio-sensing applications [3,4], e.g., biosensors (glucose, mechanical stress, magnetic field) [3,4], anticancer therapies [5,6], as well as for flexible electronic applications, e.g., supercapacitors [7], FET transistors [8,9], electrical wires [10], or as active elements in mechanical energy harvesters [11]. Well reduced fibers with flake ordered composition are reaching superior physical properties with electrical conductivity of σ = 10^6^ S·m^−1^, thermal conductivity of 1557 W·m^−1^·K^−1^, tensile strength of 1.9 GPa, and a Young’s modulus of 309 GPa [12]. A stronger connection between flakes changes the flake arrangement and alters the above-mentioned properties in comparison to previously studied graphene oxide structures formed by hydrothermal methods [2,13]. Their electrical conductivity strongly depends on the quality of the reduction process and flake ordering and can be described by variable range hopping models [14,15].

In this article, we were interested in the change of magnetic, mechanical, and electric properties of graphene oxide fibers prepared via the hydrothermal method with additional doping by divalent Ca^2+^ ions. The addition of Ca^2+^ in a coagulation bath while fibers are formed during a wet spinning method results in increased flakes tendency for the formation of a denser network with Calcium ions acting as bridges between the individual flakes [16]. This method is one of the three known among the dry spinning method [17,18] and hydrothermal method (used here) [2] for the preparation of micro-fibers. In particular, we recently showed [13] that electron paramagnetic resonance (EPR) is an effective method that allows distinguishing multiple paramagnetic contributions of magnetic susceptibility, e.g., Pauli (conduction electrons) and Curie (localized paramagnetic centers, e.g., sp^3^ defects), and could be used to study the internal ordering of flakes. The source of the EPR signal, among different carbon materials, is related to the conduction electrons, e.g., anthracite [19], or localized paramagnetic states on the surface or edges, e.g., GO [20] or graphene [21]. However, most of the aforementioned centers are present in different samples, due to preparation processes, making the synthesis method and ion doping a crucial aspect to evaluate.

Graphene-based samples can show complicated magnetic behavior due to zigzag edges [22,23], which can give rise to the edge states magnetism and basal-plane sp^3^ defects formed by -OH groups (~1–1.2 µ_B_) and adatom-induced magnetism [24,25]. We will discuss the Ca^2+^-doped fibers and compare the results to different carbon samples previously reported in the literature: graphene [21,26], GO in the form of aerogels [13,20], as well as with other carbon materials [27,28] which magnetic properties depend on particle size [29]. We will finally show the competitive mechanical and electrical response of the fibers, as well as the suitability of Ca^2+^-doping for tuning physico-chemical properties in reduced graphene oxide (rGO) fibers.

## 2. Materials and Methods

### 2.1. Graphene Oxide Preparation

Graphene oxide was purchased from the NANOPOZ company (Poznań, Poland). Flake diameters in the range of 0.9–46 µm, with a maximum number of flakes with a diameter of ~2 µm were obtained, the same as in previous reports [2]. Briefly, flake size distributions were obtained by manually measuring sizes using ImageJ^®^ from the sizes of around 200 flakes from multiple scanning electron microscopy images.

### 2.2. Reduced Graphene Oxide Fiber Preparation

**Figure 1:**
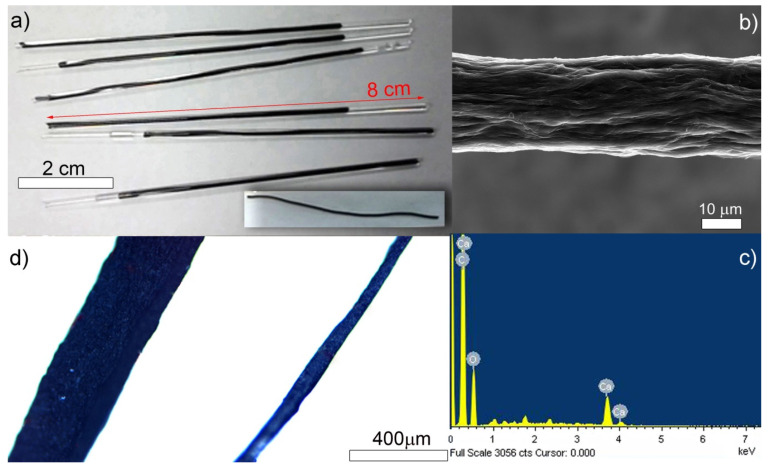
(**a**) Hydrogel in tubes after hydrothermal synthesis; (inset) fiber taken out for drying in ambient conditions (scale 1:1 with main image, glass tube length 8 cm), dried fiber is too small to be visible in this scale; (**b**) SEM micrograph of partially reduced graphene oxide fibers ~60 µm diameter; (**c**) energy dispersive X-ray spectroscopy (EDX) plot of the fiber; (**d**) optical microscope image of partially dried (~300 µm) and fully dried (~40–100 µm) diameter fibers.

GO suspension of 4 mg/mL was injected to the opened at both sides’ capillaries (with inner diameter of 0.6 mm), then sealed by melting, placed in autoclave and heated for 2 h at 180 °C, where the suspension coagulated and formed a hydrogel (Figure 1a). The hydrogel was left to dry in air at 21 °C (Figure 1a, inset). The preparation method was previously described when preparing partially reduced graphene oxide aerogels [2], except here the container was changed to Calcium containing capillaries. After the formation of xerogel, final drying was performed at 70 °C for 48 h (without further annealing at higher temperatures).

### 2.3. Scanning Electron Microscopy (SEM) and Energy Dispersive X-ray Spectroscopy (EDX)

EDX measurements were performed at 5 kV. The preparation of cross-sections for SEM analysis was carried out using a focused ion beam system JIB-4000 (JEOL) with Ga^+^ ions. The current and voltage of the ion beam during preparation were 23 pA to 60 nA and 30 kV, respectively.

### 2.4. Vibrating Sample Magnetometry (VSM)

DC magnetic susceptibility measurements were performed using the VSM Quantum Design PPMS system in a temperature range of 4 to 300 K, using a maximum applied magnetic field of ±1 T. Zero-Field-Cooled (ZFC) and Field-Cooled (FC) lines were taken for magnetic field 0.3 T (3 kOe) in a temperature range from 4 to 300 K. The value of the magnetic field was selected in order to easily compare it with the EPR measurements.

### 2.5. Electron Paramagnetic Resonance (EPR)

The spectroscopic continuous wave (CW) EPR measurements were performed with a RADIOPAN SE/X-2547 (9 GHz) spectrometer equipped with a RCX661A TM110 resonator and an Oxford CF935 cryostat allowing measurements in a temperature range of 4.2–300 K. The modulation amplitude was 0.05 mT, the microwave power was 11.38 mW (without saturation effects), and the microwave frequency was recalculated for each measured point to match exactly 9 GHz. The number of points per spectra: 1024, accumulations: 2, time per one point: 120. The number of spins was estimated by a direct comparison method with DPPH standard which was earlier calibrated with a primary standard—copper sulphate pentahydrate monocrystals. The EPR relaxation measurements were conducted with a Bruker ELEXSYS E580 EPR Spectrometer equipped with an EN4118X-MD4 resonator in a temperature range of 5–100 K. Field-sweep echo-detected (FSED) spectra were obtained from X-band pulse experiments. The shot repetition time was set at 186 µs, π/2 pulse was set to 20 ns for T_m_, T_2_, and FSED measurements, and 34 ns for inversion magnetization experiments to obtain the maximum magnetization inversion.

### 2.6. Electric Conductivity Measurements

Room temperature electric conductivity measurements were performed with a 4-point method using Keithley 2636 B SMU. The distance between the voltage electrodes was 5 mm, and the diameter of rGO fiber used was d = 50 µm.

### 2.7. Nanoindentation

The uniaxial tensile tests of rGO fibers were performed using the Agilent T150 UTM tensile machine. Fibers with a typical diameter of 91 ± 13 µm and a length of 4.5 mm were glued to the supporting frame and analyzed by the scanning electron microscope (Quanta FEG 250, FEI) to determine their dimensions—each of the fifteen fibers’ diameters were measured at five sites. The analysis allowed identifying defects in fibers’ morphology, like kinks or bend/displacement. Such defects correlate with lower tensile strength measured afterwards by the means of a uniaxial tensile test. Extension for each fiber was realized with a strain rate of 10^−4^ s^−1^ to enable a slow relaxation of fibers during the measurement. Nanoindentation tests were performed using a Triboindenter (TI950 Hysitron) with a Berkovich tip. Mechanical values were extracted using the Oliver–Pharr method [30]. Fibers were immobilized on metallic plates by commercially available silver paste. Samples were investigated at the maximum load of 1000 µN using a single signal, partial load-displacement function, and nano dynamical mechanical analysis (nanoDMA).

## 3. Results and Discussion

### 3.1. Fabrication of Reduced Graphene Oxide (rGO) Fibers

Initially, we injected a solution of 4 mg/mL graphene oxide flakes inside capillaries containing calcium ions (Figure 1a). We then subjected such capillaries to a hydrothermal treatment and then dried them out on the Teflon tape (Figure 1a, inset). The fibers were left in an ambient atmosphere for drying, which resulted in the collapse of the hydrogel pore structure and led to the formation of xerogel with a shape resulting from the capillary container, initially just after drying of about 600 µm diameter, decreased after a full drying cycle to ~50 µm (Figure 1b and Figure S1, Supporting Videos 1 and 2), and typical length decreasing from ~8 cm to ~5 cm. Further drying at 70 °C (48 h) removed remaining moisture/H_2_O from the structure but did not influence the shape or physical dimensions of the obtained fibers. The post-hydrothermal drying treatment leads to 9–10 times shorter diameters and 20–30% shortened lengths with final surface roughness Rq equal to 62.8 ± 3.3 nm in a reproducible manner, as reported previously [31].

**Figure 2:**
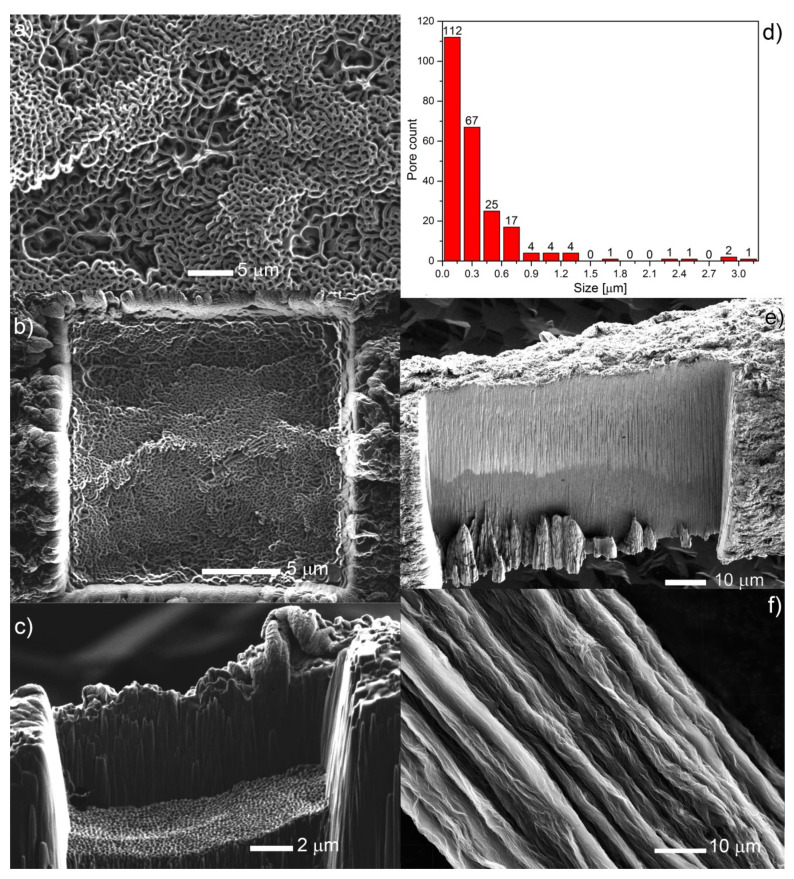
Focused ion beam images: (**a**) inside of the milled square from (**b**); (**b**) milled with gallium ions square on side of the fiber-view in the axis of Ga stream direction; (**c**) side view of a milled fiber; (**d**) pore sizes distribution taken from (**a**); (**e**) milled fiber-view perpendicular to Ga stream direction; (**f**) SEM micrograph of the fibers surface.

The surface of the fabricated fibers under scanning electron microscope (SEM) shows partially reduced graphene oxide flakes lying on each other in a co-axial arrangement (Figure 1b and Figure 2f). The reduction protocol is the same as used in our previous studies, thus we expected the produced fibers to contain the same defects [13,31]. The EDX (Figure 1c) analysis of the fibers was done at a relatively low acceleration voltage (5 kV) reducing the depth of penetration into the sample. The analysis was made for the three main elements in multiple spots (3 × 8 grid) and showed the average elemental fiber composition of C: 62, O: 32, and Ca: 6 wt.% (C/O = 1.94). As expected, quantitative maps of C, O, and Au show roughly homogeneous carbon and oxygen distribution over the entire fiber (Figure S2).

Due to obvious limitations of the SEM technique in assessing internal structure of partially reduced graphene oxide fibers, focused ion beam milling (FIB) was used. It allowed the visualization of the pseudo-porous structure inside the fiber (Figure 2a–c and Figure S4). The structure located inside the rectangular milled square in Figure 2b and presented as magnified in Figure 2a was used for the estimation of the pore surface to the surface of the selected square. Surface porosity is defined as the ratio of the overall pore area in the square to the entire square surface. The surface porosity across multiple images on average comes to ~20%. It should be mentioned that the ion beam could damage the pore structure and this data should be treated with caution, as coming from pseudo-pore structure. The porous structure is visible only when the ion beam irradiates the surface perpendicular to it, while in the other case, the flat surface is visible (Figure 2f). Figure 2d shows a pore size statistic made inside of the rectangular milled element visible in this image. The distribution’s profile can be approximated by the lognormal function. The mean pore size is 360 ± 445 nm (from 240 elements). The minimum pore diameter is 50 nm and the largest is 3150 nm.

### 3.2. Electrical Properties

**Figure 3:**
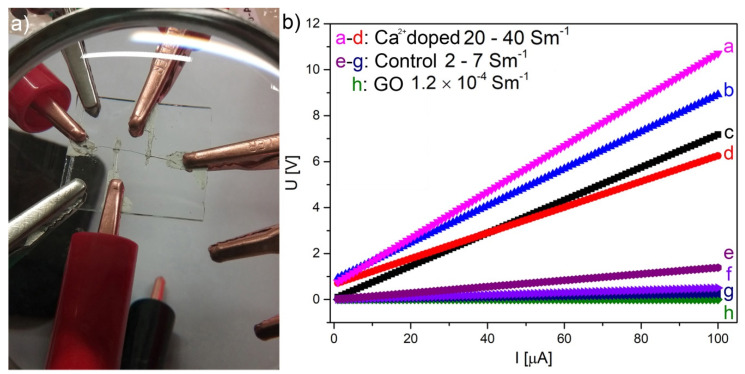
(**a**) Measurement setup 4-point method; (**b**) U = f(I) plot for four reduced graphene oxide (rGO) fibers with Ca^2+^ (a–d), four control rGO fibers without Ca^2+^ (e–g), and for graphene oxide (GO) layer made from initial suspension (h).

Improved, due to Ca^2+^-doping, layer stacking is visible in electrical measurements (DC). Current vs. voltage plot shows a linear relationship and a specific conductivity in the order of 20–40 S·m^−1^, while undoped it is in the range of 2–7 S·m^−1^ (Figure 3, Figures S5 and S6). It is known that electrical conductivity measured on a single GO flake is in the order of 0.05–2 S·m^−1^ [32], while for a fully reduced graphene oxide aerogel it is reaching 528 S·m^−1^ [33]. Our previous results obtained for partially reduced aerogels showed a conductivity of 1.7 S m^−1^ [13]. For comparison, conductivity of dried GO straps (known as GO paper) is in the order of 1.2 × 10^−4^ S·m^−1^ (Figure S5). The electrical conductivity in such systems is caused by a variable range hopping with the highest energy necessary for hopping between the flakes ($\sigma \sim e^{-{\left(T_{0}\right)}^{\frac{1}{2}}}$). Electrostatically assembled inter-flake links by Ca^2+^ decrease the energy necessary for this process by modification of the structural arrangement, causing a stronger overlap of the flakes. This mechanism can be found in systems were conductive particles are randomly connected to each other [34] with an energy barrier located in between the flakes [35]. DC measurements performed by Rani et al. [36] on compressed powders of graphite, expanded graphite, reduced graphene oxide, and carbon nanotubes showed the increase of conductivity with the increase of pressure which was explained by forming of effective contacts between grains [37,38].

### 3.3. Mechanical Properties

**Figure 4:**
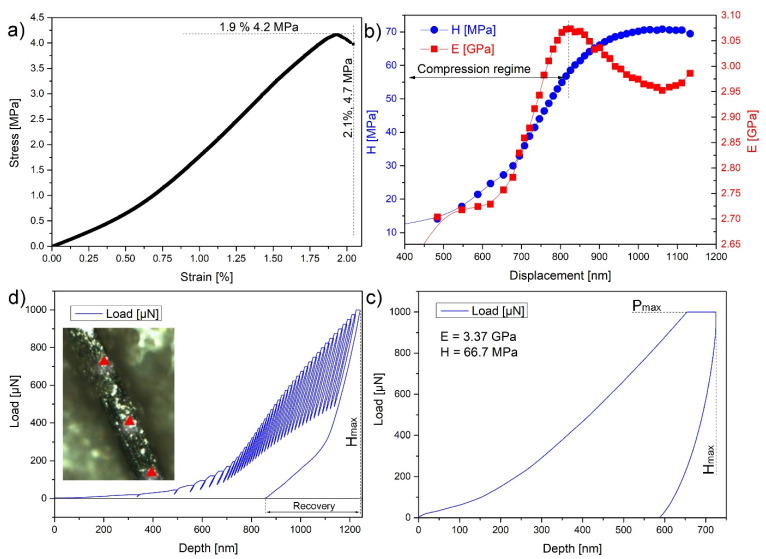
(**a**) Tensile stress–strain curve of typical partially reduced graphene oxide (prGO) fiber; (**b**) mechanical values extracted from partial load/unload test; (**c**) load vs. displacement plot of a representative indent showing maximum load (P_Max_) and maximum displacement (H_Max_); the compressive regime is marked in dashed lines; (**d**) elastic recovery of the fibers after maximum deformation (inset shows the representative indentation places).

Pristine reduced graphene oxide fibers due to having a collapsed pore structure in comparison to aerogels are much more resistant to damage. Individual performance depends on the size of the graphene flakes used, as well as their arrangement during drying and reduction [10]. To characterize the basic mechanical properties, the ultimate tensile strength was measured (Figure 4a). For the tensile strength measurements, all fiber diameters were estimated by previous SEM investigation to find the average pore diameter, which later was used as a normalization parameter (Figure 4a). The average fiber diameter taken for this measurement was 91 ± 13 µm and the length of the obtained fibers reached 5 cm. Only the fibers exhibiting proper morphology were evaluated (some omitted fibers are shown in Figure S7).

The stress–strain curve of rGO fibers indicates the brittle nature of the material without a significant plastic deformation region. The elasticity modulus calculated from the linear segment of the stress–strain curve was 360 ± 200 MPa. The fracture occurs at strain 2.1% ± 1.1%, corresponding to the stress of 4.7 ± 1.4 MPa. The value of fracture stress is about one order of magnitude lower than in the case of carbon nanofibers reinforced by GO [39] or poly(lactic acid)/GO/stearic acid composites [40], which is due to reinforcing fillers (nanotubes). Nanoindentation tests performed on the samples showed similar mechanical values to the ones reported in the literature for porous fibers, E = ~3.3 GPa and H = ~66.7 MPa. The lack of recovery on the single indentation tests (Figure 4c) suggests the highly plastic surface of the fibers, which is congruent with the SEM images and studies after FIB milling. Depth studies (Figure 4d) show the compressive regime of the nanofibers, above ~800 nm, which accounts for the plastic deformation of the sample. After that point, elastic response is observed with a 40% recovery, which is attributed to the porosity of the samples. Results for pristine fibers on the mechanical analysis reported elsewhere, are in proximity to the results obtained here: ultimate tensile strength of 29.4 MPa at 8.6% of elongation [41]. Fibers obtained by the dry spinning method show some spread of parameters: tensile strength is in the rage of 39.2–1450 MPa, elongation is in the rage of 1.1–20%, and a Young modulus of 1.9–282 GPa [42,43].

### 3.4. Magnetic Properties

Due to the different sources of a magnetic signal in plain defects ad-atoms or edge dangling bonds (edge states) the magnetism can show a complicated character. Ferro- and antiferromagnetism were theoretically predicted in graphene [44,45]. Moreover, the presence of vacancies (sp^3^ defects) caused by irradiation [46,47,48,49] or surface doping with hydrogen H^+^ ions and fluorine in the form of XeF_2_ molecules [50,51,52,53,54], or adatoms like -OH groups [24,55,56,57,58,59], moisture [35] or contamination by Mn^2+^ [60] or Fe^2+^ ion, can be source of additionally appearing exchange interactions [61,62,63,64,65]. Above that, an interplay between localized (point defects) and delocalized (conduction electrons) paramagnetic centers take place which depends on the particle size, and according to Ćirić et al. [66,67], the EPR signal comes from conduction electrons for large flakes and from localized centers for smaller than 1 μm^2^ flakes. Smaller particles (<30 nm) which have more defects, and for this reason they are more reactive, anchor 6.6 times more iron ions than larger, micrometre sized particles on the edges [60].

**Figure 5:**
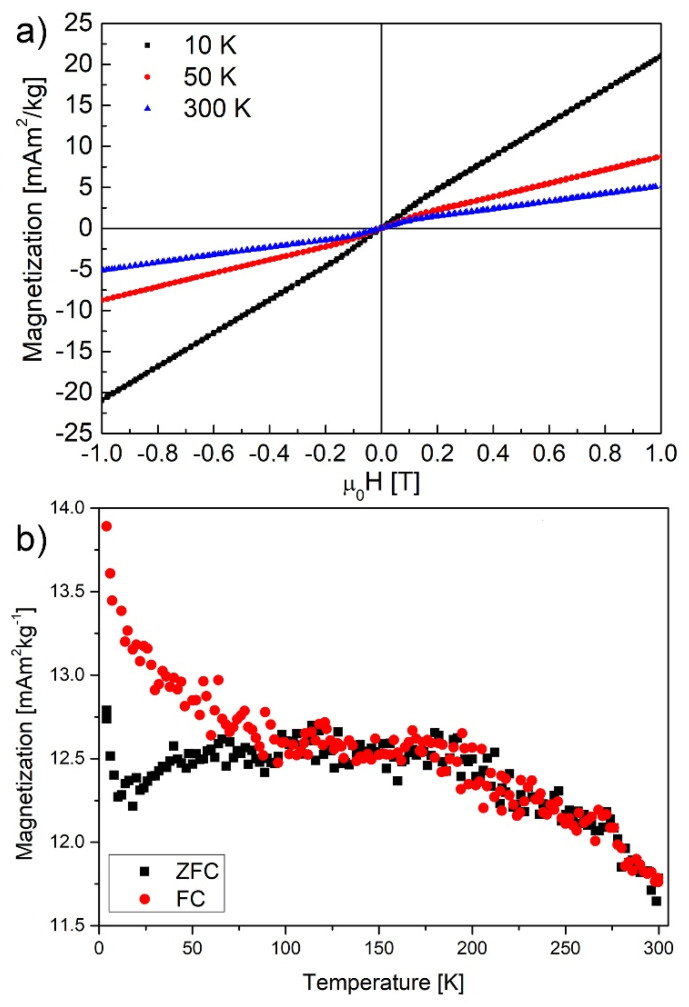
(**a**) Magnetic moment vs. magnetic field dependence recorded in the range of ±1 T for 10, 50, and 300 K; (**b**) Zero Field Cooling (ZFC) and Field Cooling (FC) curves obtained in 3 kOe in a temperature range of 5–300 K of reduced graphene oxide fibers.

Magnetization vs. magnetic field dependence of partially reduced graphene oxide fibers measured by vibrational magnetometer are in the range of ±1 T (10 kOe) (Figure 5a). As expected for paramagnetic sample, saturation magnetization grows with decreased temperature. The Zero Field Cooling (ZFC) and Field Cooling (FC) curves show differences in temperatures below 100 K indicating weak ferromagnetic interactions. The field of 3 kOe (Figure 5b) was chosen to easily compare the results with those from EPR in which the resonance signal was recorded at 9 GHz.

**Figure 6:**
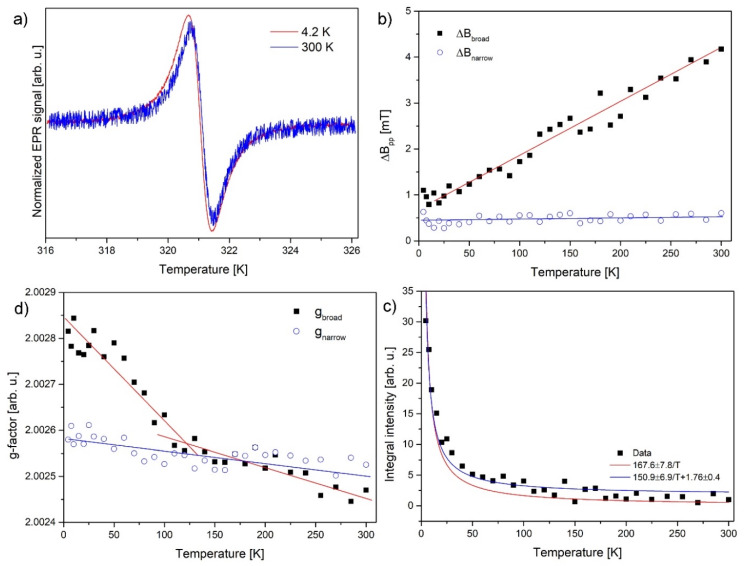
(**a**) Normalized by amplitude electron paramagnetic resonance (EPR) signals recorded at 4.2 and 300 K; (**b**) peak to peak linewidths vs. temperature for two components; (**c**) EPR integral intensity vs. temperature (red and blue curves fits explained in text); (**d**) g-factors vs. temperature for two components (straight lines are only guidelines for eyes).

EPR spectroscopy shows a single signal at a g-factor of 2.0025 (300 K) which could be decomposed to two Lorentzian lines (Figure 6a). Normalized to 300 K, the EPR intensity obeys the Curie low, approximated with the equation $\frac{167.6\;\pm \;7.8}{T}$ reaching in 4.2 K value of 30. On the other hand, better fits could be achieved by the addition of a constant positive magnetic susceptibility, approximated by the equation $\frac{167.6\;\pm \;7.8}{T}+1.76\pm 0.4,\;$which can be further interpreted as additional contribution of Pauli paramagnetism stemming from conduction electrons (Figure 6c). The presence of both localized and delocalized centers in different proportions was already shown in GO [66], reduced GO similar carbon material: Panich et al. [64], Shames et al. [68], Augustyniak-Jabłokow et al. [27].

The total number of spins was estimated as $3\times 10^{18}$ spins/g. Figure 6b,d shows the temperature dependences of g-factors and linewidths. The above g-factor is of a free radical in the entire temperature range indicating small positive spin–orbit coupling, as expected for radicals or non-interacting sp^3^ defects located on the flakes surface. The difference in g-factors of both components, which become significant below 100 K, cannot be directly explained by ZFC/FC curves in Figure 5b. It is usual that EPR signals in carbon samples can be decomposed into two lines, further changes in g-factors are small, and differences appear naturally when having different signal sources. There is no premise in EPR results presented in Figure 6, which could explain the bifurcation in ZFC/FC.

**Figure 7:**
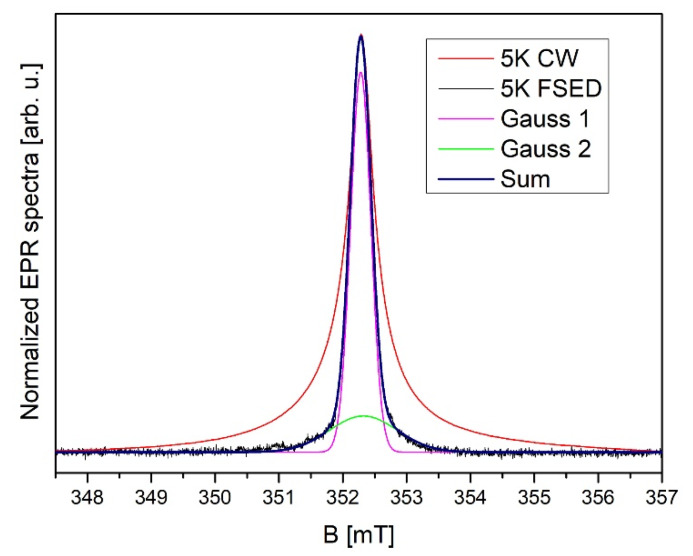
Comparison between continuous wave (CW)-EPR and field-swept echo-detected spectrum (FSED) and its decomposition into two components at 5 K.

Pulse EPR measurements confirm the presence of two lines-species in the sample (Figure 6). Two lines are visible in a field-swept echo experiment, suggesting inhomogeneous line broadening in both cases (Figure 7). Notice that the broader CW-EPR signal is shown for comparison. The lines have widths of 1.06 mT and 0.32 mT. The integral intensity of the first line is larger by a factor of 3.2.

**Figure 8:**
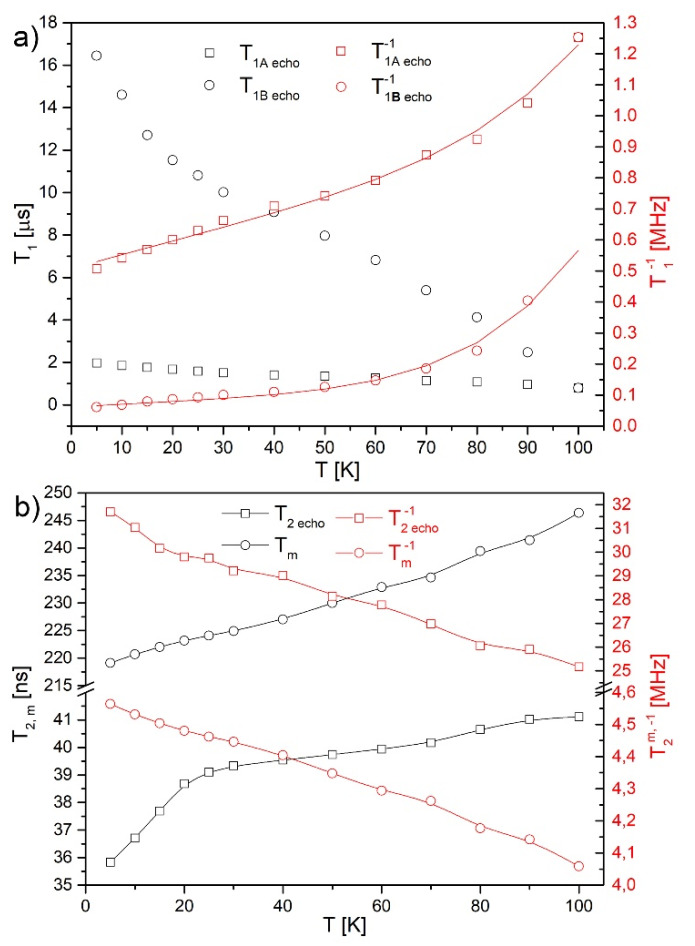
(**a**) Spin–lattice (T_1_), and (**b**) spin–spin (T_2_) and phase memory relaxation times, with fits, and their rates recorded from echo inversion as a function of temperature.

Further, the spin–lattice T_1_, phase memory time T_m_, and spin–spin relaxation time T_2echo_^*^ were recorded in the temperature range of 5–100 K (Figure 8). The spin–lattice time can be decomposed into two components, which rates at 5 K have 0.5 MHz (2 µs) and 0.06 MHz (16.6 µs), increasing with temperature to 1.25 and 0.6 MHz at 100 K, respectively (Figure 8a). The relaxation process is dominated by a direct process until around 70 K (linear function) after which a higher order Raman relaxation process becomes more dominant. Both radicals can be described by equations${\;T}_{1}^{-1}={\;A}_{0}+A_{1}\times T\;+A_{2}\times T_{5}$, where the first component is temperature independent and not related to the relaxation processes, A_1_ is the direct Raman relaxation, and A_2_ is multi phonon Raman relaxation (Table 1). In comparison, dried GO paper [20] showed spin relaxation T_1_ time of 52 ms at 5 K, while here it is 17 µs (T_1_^−1^ was 535 Hz at 100 K). The difference lies in the oxygen reduction process, which produces a larger number of surface defects and causes 3000 times faster electron spin relaxation. Moreover, a direct relaxation process in GO paper dominates up to 100 K, which as it seems can be treated as an indicator of a small number of defects. It seems that the reduction process increases the number of surface defects and by that the relaxation rate, which further starts to be dominated by a higher order Raman processes already at lower temperature.

In the wider picture, electron relaxation studies performed on other carbon materials show following results: Panich et al. reported graphene oxide heavily doped with Mn^2+^ ions showing T_1_ (from saturation measurements): <10 ns, T_1_^−1^ = 100 MHz [64], for nanographites doped with Fe^2+,3+^ T_1_ < 10^−9^ s [60], Rao et al. showed graphene nanoribbons two spin lattice relaxation processes: T_1_^−1^ ~0.014 MHz and 0.1 MHz [69]. For graphene, T_1_^−1^~T_2_^−1*^~3 MHz was reported at 100 K [21] (and 0.7 kHz at room temperature to 1.45 kHz at helium temperature). In partially reduced graphene oxide (prGO) with an average flake size of ~2 µm the relaxation times were 17.5 MHz (T_1_^−1^ = T_2_^−1^) for which the reduction caused the increase of the spin–lattice relaxation rate T_1_^−1^ by a factor of ~5.8 [13]. For Ca-doped fibers, the T_1_^−1^ is lower at 100 K around 1.25 MHz, compared to undoped fibers mentioned above at 17.5 MHz.

The spin–spin times $T_{2}^{*}$ were fitted by a single exponential function$\;V\left(t\right)=A\times \exp\;\left(-\frac{t}{T_{2}^{*}}\right)$ from the free induction decay. The phase memory time was evaluated at each temperature from the echo intensity decay with the increase of the dwell time between two pulses as well as the mono-exponential function used of the form$\;V\left(2\tau \right)=A\times \exp\;\left(-\frac{2\tau }{T_{m}}\right)$. T_1_ is much longer than T_m_, 27 times longer in 5 K, which suggests that T_m_ is dominated by molecular motions.

The phase memory and spin–spin relaxation rates grow with the increase of temperature, as suggested by the broadening of the EPR line (Figure 8b). The spin packets are ~12.2 times narrower than the line width (T_2_^*^ = 35.8 ns, T_m_ = 219.1 ns). The motional narrowing of the spin packets (decrease of the relaxation rate) corresponds to the decrease of the EPR line (T_2_^−1*^), which may be caused only by motional narrowing of spin packets. As far as it goes for the tendency, the decrease of the phase memory relaxation rate is similar to the one observed in graphene oxide [20] and it seems natural for this sample. The difference in the case of GO lies in the presence of oxygen rich groups and their re-orientations under the influence of a magnetic field. This process can be caused by motion of the centers due to the increase of temperature or by an exchange interaction between individual resonance frequencies of the paramagnetic centers.

## 4. Conclusions

In summary, we used calcium containing capillaries during hydrothermal fabrication of graphene oxide dispersion to form Ca^2+^ ions doped reduced graphene oxide fibers. Formed fibers appeared to have lateral alignment of GO flakes around the fiber axis with stronger inter-flake connections developed by cross-linking points of divalent Calcium ions. Doped fibers exhibit tensile strength in the order of 4.7 MPa and an elastic modulus of 360 MPa. Nanoindentation tests performed on site of the fiber showed the following values: E = ~3.3 GPa and H = ~66.7 MPa. The changed flake ordering caused by Ca^2+^ ions increased electrical conductivity and decreased the electron spin–lattice relaxation rate $T_{1}^{-1}$. The electron spin relaxation rate increased in comparison to the results obtained previously for hydrothermally produced GO paper, whereas the specific conductivity also increased to ~20–40 S·m^−1^ (compared to 2–7 S·m^−1^ undoped rGO fibres/films and 1.2 × 10^−4^ S·m^−1^ for GO paper). The sample is paramagnetic, exhibiting two EPR signals resulting from two different sp^3^ defects (-OH groups), other structural defects, as well as conduction electrons. The relaxation T_1_ rates can be described by a direct process and multiphoton Raman scattering for temperatures reaching 100 K. Thermal reduction increases the T_1_ relaxation rate. Removing the oxygen surface groups makes the formation of local phonons easier, which ends with a T^5^ relaxation order dependence.

Our results point to an easy way of tuning the properties of hydrothermally formed graphene oxide-based structures by replacing containers with calcium, which could bridge the flakes by inter-connections and enhance the flake–flake interactions, thus forming more conductive fibers for multiple applications, including electronics, bio-electronics, and cell-culture applications.

## Figures and Tables

**Table 1:** Components of different relaxation processes for two paramagnetic centers.

T_1A_^−1^	T_1B_^−1^	T_1GO_^−1^ [20]
A_0_ = 0.51 s^−1^	A_0_ = 0.06 s^−1^	
A_1_ = 4.4 × 10^−3^ K^−1^·s^−1^	A_1_ = 9.0 × 10^−4^ K^−1^·s^−1^	A_1_ = 4.7 × 10^−4^ K^−1^·s^−1^
A_2_ = 2.8 × 10^−11^ K^−5^·s^−1^	A_2_ = 4.2 × 10^−11^ K^−5^·s^−1^	A_2_ = 6.6 × 10^−9^ K^−5^·s^−1^
*R*^2^ = 0.9995	*R*^2^ = 0.9957	

## Supplementary Materials

The following are available online at https://www.mdpi.com/2079-4991/10/5/957/s1, Supporting information—document file; Figure S1: SEM image of single fiber; Figure S2: (top left) SEM image of a fiber quantitative EDX map of (top right) carbon, (bottom right) oxygen, (bottom left) gold substrate; Figure S3: FIB images of gallium ion grinded GO fiber; Figure S4: FIB image and mask for pore area count; Figure S5: DC conductivity measurements performed at 21 °C on GO strap and Ca^2+^-doped and partially reduced fiber after drying; Figure S6: Electrical conductivity measured with the four-point method at room temperature; Figure S7: SEM images of the prGO fibers: (a) correct flake arrangement, (b) kinks defect, and (c) bend/displacement defect; Figure S8: Microscopic image of fiber xerogels, diameter of 40–100 µm; Videos S1 and S2: Time-lapse record of a drying process in which hydrogel transforms into a collapsed prGO structure called a xerogel.

## References

[1] Colburn A., Wanninayake N., Kim D.Y., Bhattacharyya D. (2018). Cellulose-graphene quantum dot composite membranes using ionic liquid. J. Membr. Sci..

[2] Tadyszak K., Majchrzycki Ł., Szyller Ł., Scheibe B. (2018). Preparation and characterization of partially reduced graphene oxide aerogels doped with transition metal ions. J. Mater. Sci..

[3] Cayuela A., Soriano M.L., Carrillo-Carrión C., Valcárcel M. (2016). Semiconductor and carbon-based fluorescent nanodots: The need for consistency. Chem. Commun..

[4] Benítez-Martínez S., Valcárcel M. (2015). Graphene quantum dots in analytical science. TrAC Trends Anal. Chem..

[5] De S., Patra K., Ghosh D., Dutta K., Dey A., Sarkar G., Maiti J., Basu A., Rana D., Chattopadhyay D. (2018). Tailoring the Efficacy of Multifunctional Biopolymeric Graphene Oxide Quantum Dot-Based Nanomaterial as Nanocargo in Cancer Therapeutic Application. ACS Biomater. Sci. Eng..

[6] Cao J., An H., Huang X., Fu G., Zhuang R., Zhu L., Xie J., Zhang F. (2016). Monitoring of the tumor response to nano-graphene oxide-mediated photothermal/photodynamic therapy by diffusion-weighted and BOLD MRI. Nanoscale.

[7] Zhao X., Zheng B., Huang T., Gao C. (2015). Graphene-based single fiber supercapacitor with a coaxial structure. Nanoscale.

[8] Kim B.J., Jang H., Lee S.-K., Hong B.H., Ahn J.-H., Cho J.H. (2010). High-Performance Flexible Graphene Field Effect Transistors with Ion Gel Gate Dielectrics. Nano Lett..

[9] Yoon S.S., Lee K.E., Cha H.-J., Seong D.G., Um M.-K., Byun J.-H., Oh Y., Oh J.H., Lee W., Lee J.U. (2015). Highly Conductive Graphene/Ag Hybrid Fibers for Flexible Fiber-Type Transistors. Sci. Rep..

[10] Xu Z., Sun H., Zhao X., Gao C. (2013). Ultrastrong Fibers Assembled from Giant Graphene Oxide Sheets. Adv. Mater..

[11] Kim S.H., Haines C.S., Li N., Kim K.J., Mun T.J., Choi C., Di J., Oh Y.J., Oviedo J.P., Bykova J. (2017). Harvesting electrical energy from carbon nanotube yarn twist. Science.

[12] Augustyniak-Jabłokow M.A., Fedaruk R., Strzelczyk R., Majchrzycki Ł. (2019). Identification of a Slowly Relaxing Paramagnetic Center in Graphene Oxide. Appl. Magn. Reson..

[13] Tadyszak K., Chybczyńska K., Ławniczak P., Zalewska A., Cieniek B., Gonet M., Murias M. (2019). Magnetic and electric properties of partially reduced graphene oxide aerogels. J. Magn. Magn. Mater..

[14] Joung D., Khondaker S.I. (2012). Efros-Shklovskii variable-range hopping in reduced graphene oxide sheets of varying carbon *sp*^2^ fraction. Phys. Rev. B.

[15] Park M., Hong S.J., Kim K.H., Kang H., Lee M., Jeong D.H., Park Y.W., Kim B.H. (2017). Electrical and thermoelectric transport by variable range hopping in reduced graphene oxide. Appl. Phys. Lett..

[16] Wu C., Wang X., Zhuo Q., Sun J., Qin C., Wang J., Dai L. (2018). A facile continuous wet-spinning of graphene oxide fibers from aqueous solutions at high pH with the introduction of ammonia. Carbon.

[17] Tian Q., Xu Z., Liu Y., Fang B., Peng L., Xi J., Li Z., Gao C. (2017). Dry spinning approach to continuous graphene fibers with high toughness. Nanoscale.

[18] Feng L., Chang Y., Zhong J., Jia D.-C. (2018). Dry Spin Graphene Oxide Fibers: Mechanical/Electrical Properties and Microstructure Evolution. Sci. Rep..

[19] Tadyszak K., Augustyniak-Jabłokow M.A., Więckowski A.B., Najder-Kozdrowska L., Strzelczyk R., Andrzejewski B. (2015). Origin of electron paramagnetic resonance signal in anthracite. Carbon.

[20] Augustyniak-Jabłokow M.A., Tadyszak K., Strzelczyk R., Fedaruk R., Carmieli R. (2019). Slow spin relaxation of paramagnetic centers in graphene oxide. Carbon.

[21] Augustyniak-Jabłokow M.A., Tadyszak K., MaćKowiak M., Lijewski S. (2013). ESR study of spin relaxation in graphene. Chem. Phys. Lett..

[22] Augustyniak-Jabłokow M.A., Maćkowiak M., Tadyszak K., Strzelczyk R. (2015). FMR evidence of stable ferromagnetic correlations at zigzag edge states in graphene. Acta Phys. Pol. A.

[23] Tadyszak K., Maćkowiak M., Augustyniak-Jabłokow M.A., Roman S. (2014). FMR evidence of ferromagnetic correlations at zigzag edge states in single-layer graphene. J. Mol. Struct..

[24] Boukhvalov D.W., Katsnelson M.I. (2011). sp-Electron Magnetic Clusters with a Large Spin in Graphene. ACS Nano.

[25] Santos E.J.G., Ayuela A., Sánchez-Portal D. (2012). Universal magnetic properties of sp3-type defects in covalently functionalized graphene. New J. Phys..

[26] Augustyniak-Jabłokow M.A., Tadyszak K., Maćkowiak M., Yablokov Y.V. (2011). EPR evidence of antiferromagnetic ordering in single-layer graphene. Phys. Status Solidi Rapid Res. Lett..

[27] Augustyniak-Jabłokow M.A., Yablokov Y.V., Andrzejewski B., Kempiński W., Łoś S., Tadyszak K., Yablokov M.Y., Zhikharev V.A. (2010). EPR and magnetism of the nanostructured natural carbonaceous material shungite. Phys. Chem. Miner..

[28] Fedaruk R., Strzelczyk R., Tadyszak K., Markevich S.A., Augustyniak-Jabłokow M.A. (2017). Effect of Rabi splitting on the low-temperature electron paramagnetic resonance signal of anthracite. J. Magn. Reson..

[29] Tadyszak K., Strzelczyk R., Coy E., Mac’Kowiak M., Augustyniak-Jabłokow M.A. (2016). Size effects in the conduction electron spin resonance of anthracite and higher anthraxolite. Magn. Reson. Chem..

[30] Pharr G.M., Oliver W.C. (1992). Measurement of Thin Film Mechanical Properties Using Nanoindentation. MRS Bull..

[31] Wychowaniec J.K., Litowczenko J., Tadyszak K. (2020). Fabricating versatile cell supports from nano- and micro-sized graphene oxide flakes. J. Mech. Behav. Biomed. Mater..

[32] Gómez-Navarro C., Weitz R.T., Bittner A.M., Scolari M., Mews A., Burghard M., Kern K. (2007). Electronic Transport Properties of Individual Chemically Reduced Graphene Oxide Sheets. Nano Lett..

[33] George G., Costas G. (2017). Graphene aerogels: A review. 2D Mater..

[34] Kaiser A.B., Gómez-Navarro C., Sundaram R.S., Burghard M., Kern K. (2009). Electrical Conduction Mechanism in Chemically Derived Graphene Monolayers. Nano Lett..

[35] Kempiński M., Florczak P., Jurga S., Śliwińska-Bartkowiak M., Kempiński W. (2017). The impact of adsorption on the localization of spins in graphene oxide and reduced graphene oxide, observed with electron paramagnetic resonance. Appl. Phys. Lett..

[36] Rani A., Nam S., Oh K.A., Park M. (2010). Electrical Conductivity of Chemically Reduced Graphene Powders under Compression. Carbon Lett..

[37] Park W., Hu J., Jauregui L.A., Ruan X., Chen Y.P. (2014). Electrical and thermal conductivities of reduced graphene oxide/polystyrene composites. Appl. Phys. Lett..

[38] Haque A., Abdullah-Al Mamun M., Taufique M.F.N., Karnati P., Ghosh K. (2018). Large Magnetoresistance and Electrical Transport Properties in Reduced Graphene Oxide Thin Film. IEEE Trans. Magn..

[39] Lin X., Liu X., Jia J., Shen X., Kim J.-K. (2014). Electrical and mechanical properties of carbon nanofiber/graphene oxide hybrid papers. Compos. Sci. Technol..

[40] Chartarrayawadee W., Molloy R., Ratchawet A., Janmee N., Butsamran M., Panpai K. (2015). Fabrication of poly(lactic acid)/graphene oxide/stearic acid composites with improved tensile strength. Polym. Compos..

[41] Uçar N., Ölmez M., Kayaoğlu B.K., Önen A., Karatepe Yavuz N., Eksik O. (2018). Structural properties of graphene oxide fibers: From graphene oxide dispersion until continuous graphene oxide fiber. J. Text. Inst..

[42] Zeng J., Liu Y., Han D., Yu B., Deng S., Chen F., Fu Q. (2018). Mechanical property enhancement of high conductive reduced graphene oxide fiber by a small loading of polydopamine. Mater. Res. Express.

[43] Xu Z., Gao C. (2015). Graphene fiber: A new trend in carbon fibers. Mater. Today.

[44] Dutta S., Lakshmi S., Pati S.K. (2008). Electron-electron interactions on the edge states of graphene: A many-body configuration interaction study. Phys. Rev. B.

[45] Wang Y., Huang Y., Song Y., Zhang X., Ma Y., Liang J., Chen Y. (2009). Room-Temperature Ferromagnetism of Graphene. Nano Lett..

[46] Yazyev O.V. (2010). Emergence of magnetism in graphene materials and nanostructures. Rep. Prog. Phys..

[47] Ney A., Papakonstantinou P., Kumar A., Shang N.-G., Peng N. (2011). Irradiation enhanced paramagnetism on graphene nanoflakes. Appl. Phys. Lett..

[48] Yazyev O.V., Helm L. (2007). Defect-induced magnetism in graphene. Phys. Rev. B.

[49] López-Sancho M.P., de Juan F., Vozmediano M.A.H. (2009). Magnetic moments in the presence of topological defects in graphene. Phys. Rev. B.

[50] Nair R.R., Sepioni M., Tsai I.L., Lehtinen O., Keinonen J., Krasheninnikov A.V., Thomson T., Geim A.K., Grigorieva I.V. (2012). Spin-half paramagnetism in graphene induced by point defects. Nat. Phys..

[51] Eng A.Y.S., Poh H.L., Šaněk F., Maryško M., Matějková S., Sofer Z., Pumera M. (2013). Searching for Magnetism in Hydrogenated Graphene: Using Highly Hydrogenated Graphene Prepared via Birch Reduction of Graphite Oxides. ACS Nano.

[52] Xie L., Wang X., Lu J., Ni Z., Luo Z., Mao H., Wang R., Wang Y., Huang H., Qi D. (2011). Room temperature ferromagnetism in partially hydrogenated epitaxial graphene. Appl. Phys. Lett..

[53] Feng Q., Tang N., Liu F., Cao Q., Zheng W., Ren W., Wan X., Du Y. (2013). Obtaining High Localized Spin Magnetic Moments by Fluorination of Reduced Graphene Oxide. ACS Nano.

[54] Kim H.-J., Cho J.-H. (2013). Fluorine-induced local magnetic moment in graphene: A hybrid DFT study. Phys. Rev. B.

[55] Sepioni M., Nair R.R., Rablen S., Narayanan J., Tuna F., Winpenny R., Geim A.K., Grigorieva I.V. (2010). Limits on Intrinsic Magnetism in Graphene. Phys. Rev. Lett..

[56] Lehtinen P.O., Foster A.S., Ayuela A., Krasheninnikov A., Nordlund K., Nieminen R.M. (2003). Magnetic Properties and Diffusion of Adatoms on a Graphene Sheet. Phys. Rev. Lett..

[57] Boukhvalov D.W. (2010). Modeling of hydrogen and hydroxyl group migration on graphene. Phys. Chem. Chem. Phys..

[58] Ganguly A., Sharma S., Papakonstantinou P., Hamilton J. (2011). Probing the Thermal Deoxygenation of Graphene Oxide Using High-Resolution In Situ X-ray-Based Spectroscopies. J. Phys. Chem. C.

[59] Mattevi C., Eda G., Agnoli S., Miller S., Mkhoyan K.A., Celik O., Mastrogiovanni D., Granozzi G., Garfunkel E., Chhowalla M. (2009). Evolution of Electrical, Chemical, and Structural Properties of Transparent and Conducting Chemically Derived Graphene Thin Films. Adv. Funct. Mater..

[60] Panich A.M., Shames A.I., Tsindlekht M.I., Osipov V.Y., Patel M., Savaram K., He H. (2016). Structure and Magnetic Properties of Pristine and Fe-Doped Micro- and Nanographenes. J. Phys. Chem. C.

[61] Tadyszak K., Rudowicz C., Ohta H., Sakurai T. (2017). Electron magnetic resonance data on high-spin Mn (III; S = 2) ions in porphyrinic and salen complexes modeled by microscopic spin Hamiltonian approach. J. Inorg. Biochem..

[62] Tadyszak K., Rudowicz C. (2017). EMR data on Mn(III; S = 2) ions in MnTPPCl complex modelled by microscopic spin hamiltonian approach. Acta Phys. Pol. A.

[63] Rudowicz C., Tadyszak K. (2017). Single magnetic 3dNadatoms on surfaces—Proper outlook on compatibility of orthorhombic zero-field splitting parameters and their relationships with magnetic anisotropy quantities. Polyhedron.

[64] Panich A.M., Shames A.I., Aleksenskii A.E., Dideikin A. (2012). Magnetic resonance evidence of manganese–graphene complexes in reduced graphene oxide. Solid State Commun..

[65] Panich A.M., Shames A.I., Sergeev N.A. (2013). Paramagnetic Impurities in Graphene Oxide. Appl. Magn. Reson..

[66] Ćirić L., Sienkiewicz A., Djokić D.M., Smajda R., Magrez A., Kaspar T., Nesper R., Forró L. (2010). Size dependence of the magnetic response of graphite oxide and graphene flakes—An electron spin resonance study. Phys. Status Solidi B.

[67] Ćirić L., Sienkiewicz A., Gaál R., Jaćimović J., Vâju C., Magrez A., Forró L. (2012). Defects and localization in chemically-derived graphene. Phys. Rev. B.

[68] Shames A.I., Felner I., Osipov V.Y., Katz E.A., Mogilko E., Grinblat J., Panich A.M., Belousov V.P., Belousova I.M., Ponomarev A.N. (2011). Closed pi-electron Network in Large Polyhedral Multi-shell Carbon Nanoparticles. Nanosci. Nanotechnol. Lett..

[69] Rao S.S., Stesmans A., van Tol J., Kosynkin D.V., Higginbotham-Duque A., Lu W., Sinitskii A., Tour J.M. (2012). Spin Dynamics and Relaxation in Graphene Nanoribbons: Electron Spin Resonance Probing. ACS Nano.

