# Supplementary material for: Tuning Properties of Partially Reduced Graphene Oxide Fibers upon Calcium Doping
Source: Nanomaterials (Basel). 2020 May 18;10(5):957. doi: 10.3390/nano10050957 (PMC7325576; doi:10.3390/nano10050957)
Supplement: Supplementary file 1 [file nanomaterials-10-00957-s001.zip › nanomaterials-785960-supple/nanomaterials-785960-supple.pdf]

nanomaterials

Article

Tuning Properties of Partially Reduced Graphene Oxide Fibers upon Calcium Doping

Krzysztof Tadyszak [1,*], Jacek K. Wychowaniec [2,3], Karol Załęski [3], Emerson Coy [3], Łukasz Majchrzycki [4] and Raanan Carmieli [5]

1 Institute of Molecular Physics, Polish Academy of Sciences, ul. Smoluchowskiego 17, 60-179 Poznań, Poland
2 School of Chemistry, University College Dublin, Belfield, Dublin 4, Ireland; jacek.wychowaniec@ucd.ie
3 NanoBioMedical Centre, Adam Mickiewicz University, Wszechnicy Piastowskiej 3, 61-614 Poznań, Poland; zaleski@amu.edu.pl (K.Z.); coyeme@amu.edu.pl (E.C.)
4 Center of Advanced Technology, Adam Mickiewicz University, ul. Uniwersytetu Poznańskiego 10, 61-614 Poznań, Poland; lukmaj@amu.edu.pl
5 Department of Chemical Research Support, Faculty of Chemistry, Weizmann Institute of Science, Rehovot 76100, Israel; raanan.carmieli@weizmann.ac.il
* Correspondence: krzysztof.tadyszak@ifmpan.poznan.pl

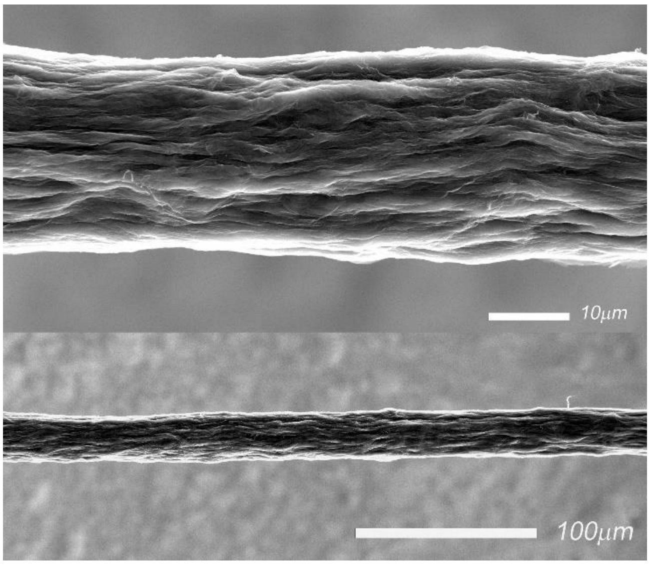

Figure S1. SEM image of single fiber.

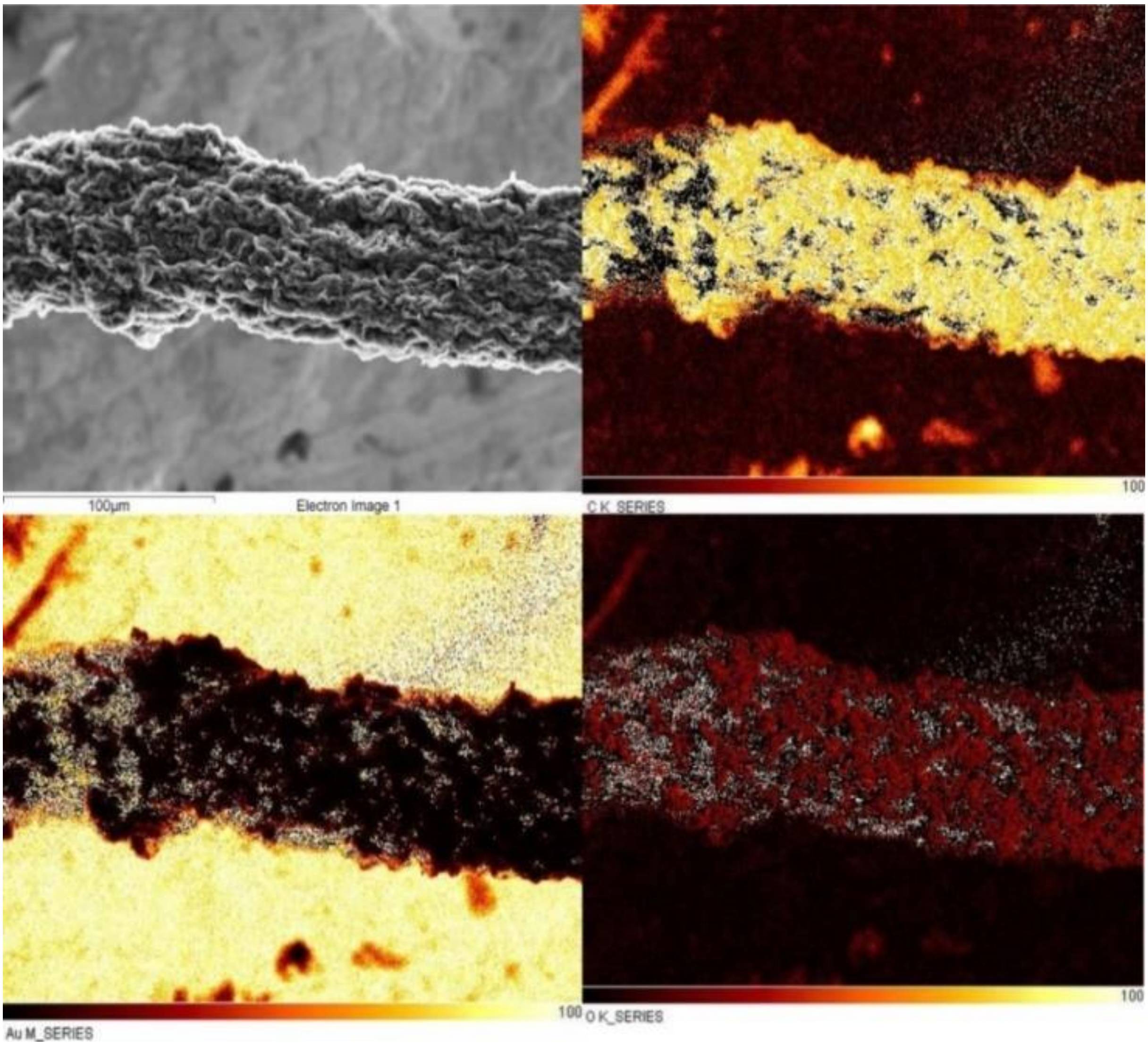

Figure S2. (top left) SEM image of a fiber quantitative EDX map of: (top right) carbon, (bottom right) oxygen, (bottom left) gold substrate.

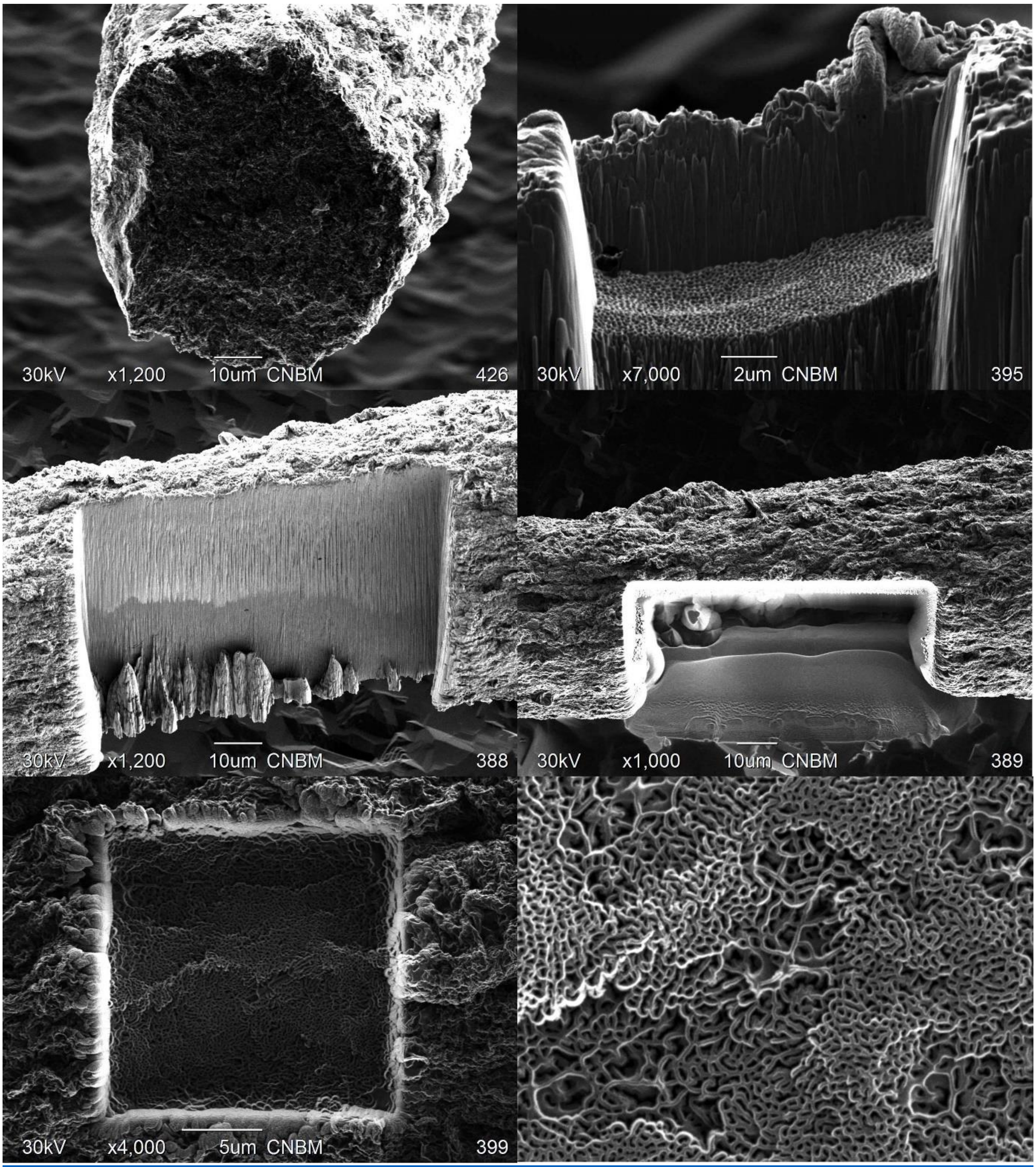

Figure S3. FIB images of gallium ion grinded GO fiber. The pseudo pore structure is visible only in the direction of the beam.

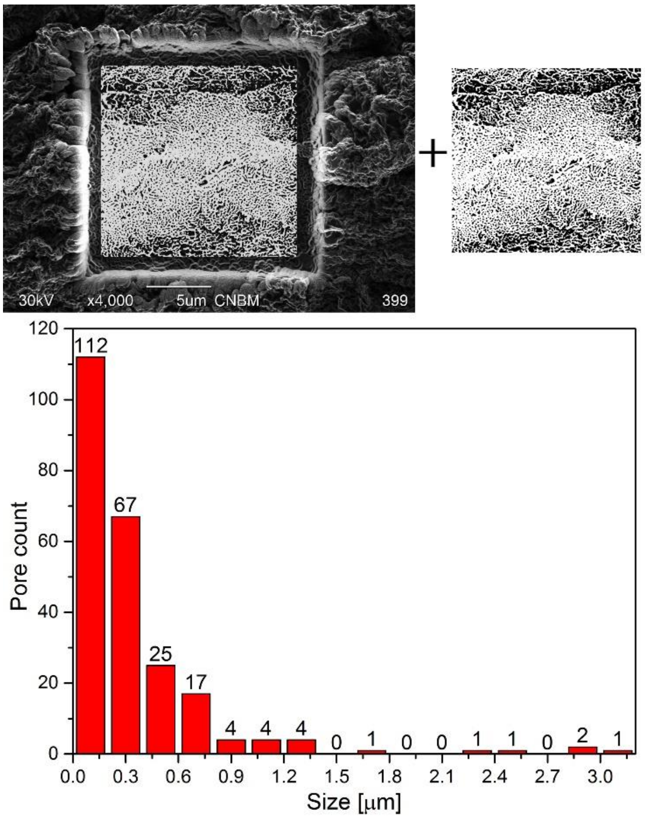

Figure S4. FIB image and mask for pore area count. Number of black pixels in the image corresponds to pore area.

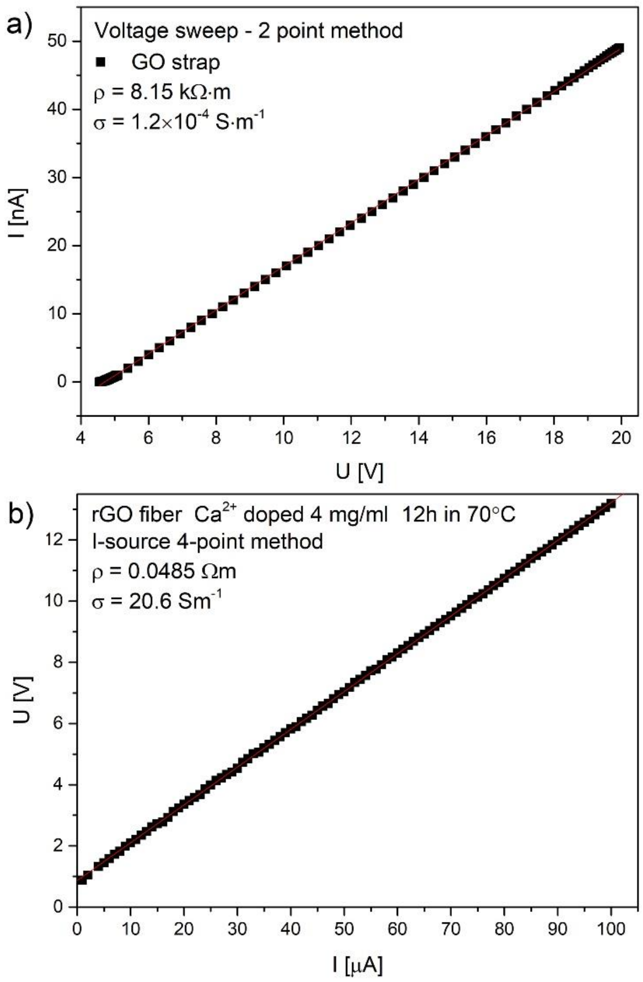

Figure S5. DC conductivity measurements performed in 21°C on GO strap and Ca^{2+} doped and partially reduced fibre after drying.

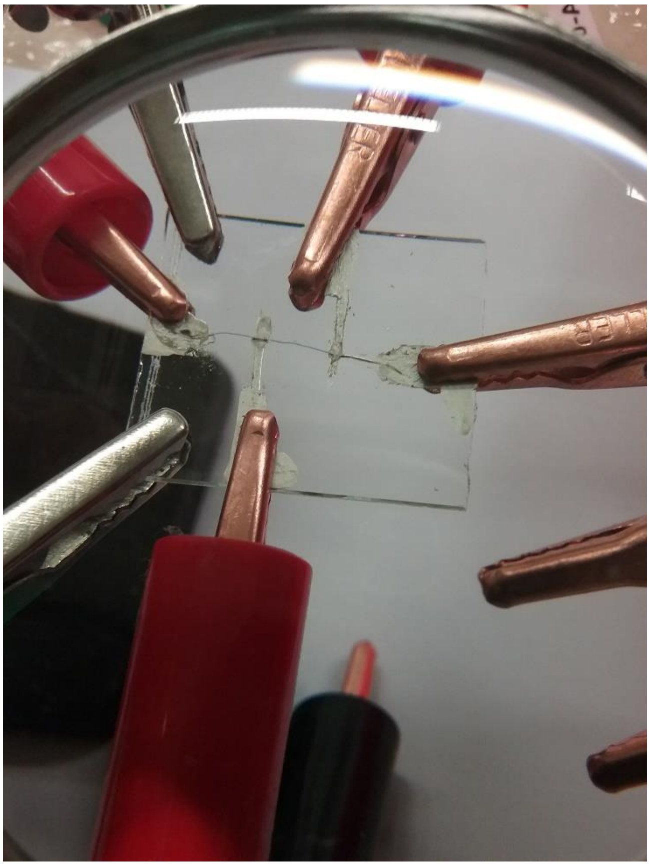

Figure S6. Electrical conductivity measured with four-point method at room temperature.

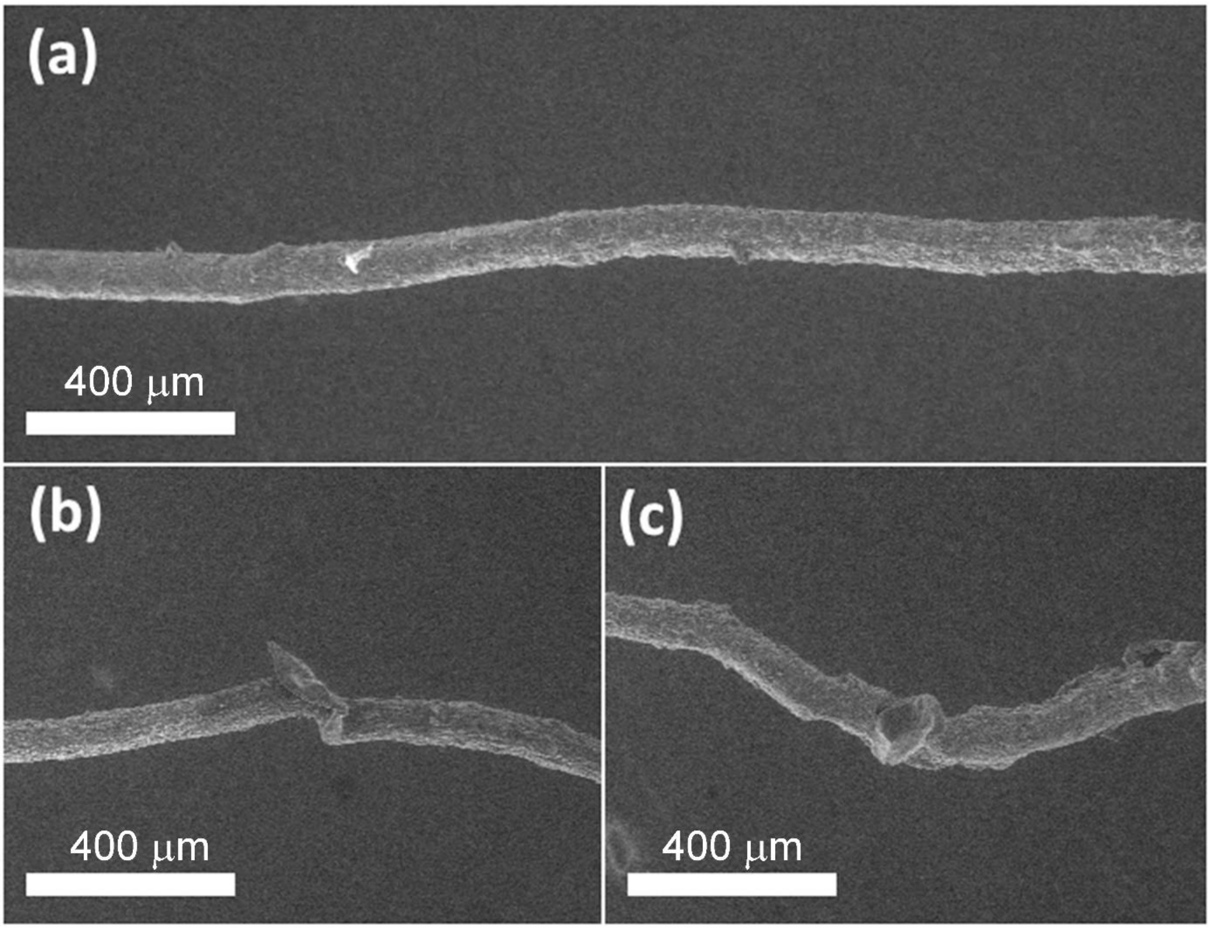

Figure S7. SEM images of the prGO fibers: **a**) correct flake arrangement, **b**) kinks defect **c**) bend/displacement defect. For the tensile strength analysis was performed only on correctly aligned fibers were used. Scalebars are 400 μm.

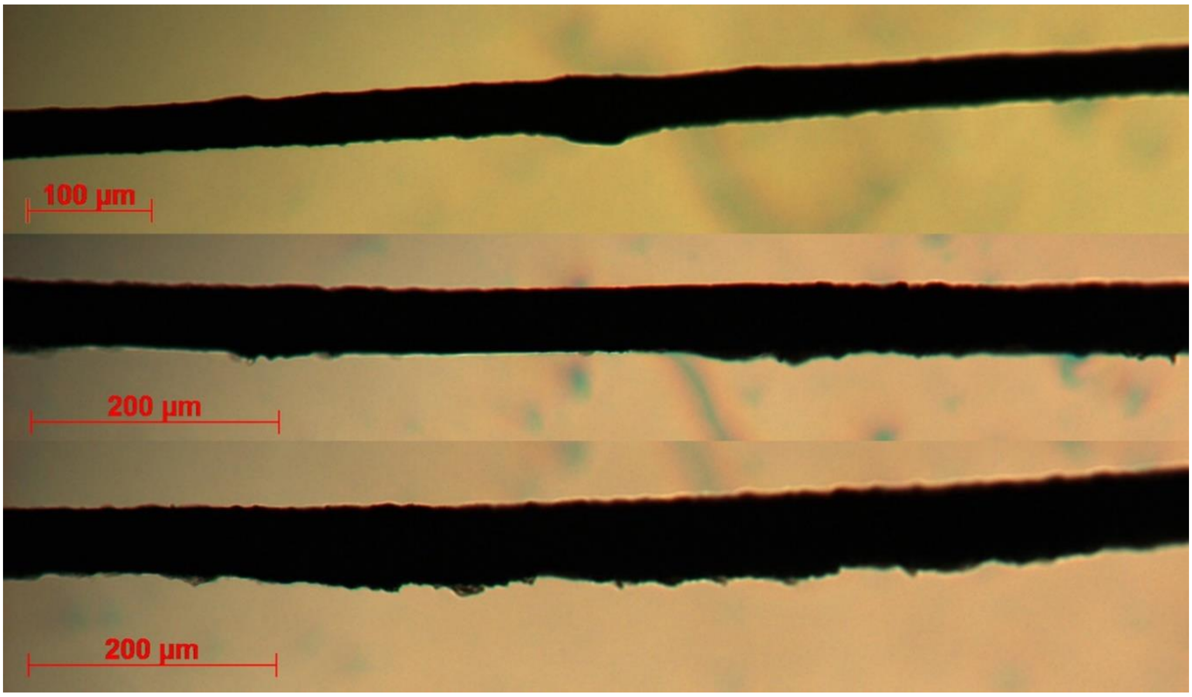

Figure S8. Microscopic image of fiber xerogels, diameter of 40–100 μm.
